# Effectiveness of Hypertension Management Strategies in SPRINT‐Eligible US Adults: A Simulation Study

**DOI:** 10.1161/JAHA.123.032370

**Published:** 2024-01-12

**Authors:** Fengdi Zhang, Kelsey B. Bryant, Andrew E. Moran, Yiyi Zhang, Jordana B. Cohen, Adam P. Bress, James P. Sheppard, Jordan B. King, Catherine G. Derington, William S. Weintraub, Ian M. Kronish, Steven Shea, Brandon K. Bellows

**Affiliations:** ^1^ Department of Medicine Columbia University New York NY USA; ^2^ Department of Medicine Mount Sinai New York NY USA; ^3^ Department of Medicine and Department of Biostatistics, Epidemiology, and Informatics University of Pennsylvania Philadelphia PA USA; ^4^ Intermountain Healthcare Department of Population Health Sciences University of Utah Salt Lake City UT USA; ^5^ Nuffield Department of Primary Care Health Sciences University of Oxford UK; ^6^ Institute for Health Research Kaiser Permanente Colorado Aurora CO USA; ^7^ Department of Medicine Georgetown University Washington DC USA; ^8^ MedStar Health Research Institute Washington DC USA

**Keywords:** blood pressure, cardiovascular diseases, hypertension, Hypertension, High Blood Pressure

## Abstract

**Background:**

Despite reducing cardiovascular disease (CVD) events and death in SPRINT (Systolic Blood Pressure Intervention Trial), intensive systolic blood pressure goals have not been adopted in the United States. This study aimed to simulate the potential long‐term impact of 4 hypertension management strategies in SPRINT‐eligible US adults.

**Methods and Results:**

The validated Blood Pressure Control–Cardiovascular Disease Policy Model, a discrete event simulation of hypertension care processes (ie, visit frequency, blood pressure [BP] measurement accuracy, medication intensification, and medication adherence) and CVD outcomes, was populated with 25 000 SPRINT‐eligible US adults. Four hypertension management strategies were simulated: (1) usual care targeting BP <140/90 mm Hg (Seventh Report of the Joint National Committee on Prevention, Detection, Evaluation, and Treatment of High Blood Pressure usual care), (2) intensive care per the SPRINT protocol targeting BP <120/90 mm Hg (SPRINT intensive), (3) usual care targeting guideline‐recommended BP <130/80 mm Hg (American College of Cardiology/American Heart Association usual care), and (4) team‐based care added to usual care and targeting BP <130/80 mm Hg. Relative to the Seventh Report of the Joint National Committee on Prevention, Detection, Evaluation, and Treatment of High Blood Pressure usual care, among the 18.1 million SPRINT‐eligible US adults, an estimated 138 100 total CVD events could be prevented per year with SPRINT intensive, 33 900 with American College of Cardiology/American Heart Association usual care, and 89 100 with team‐based care. Compared with the Seventh Report of the Joint National Committee on Prevention, Detection, Evaluation, and Treatment of High Blood Pressure usual care, SPRINT intensive care was projected to increase treatment‐related serious adverse events by 77 600 per year, American College of Cardiology/American Heart Association usual care by 33 300, and team‐based care by 27 200.

**Conclusions:**

As BP control has declined in recent years, health systems must prioritize hypertension management and invest in effective strategies. Adding team‐based care to usual care may be a pragmatic way to manage risk in this high‐CVD‐risk population.

**Nonstandard Abbreviations and Acronyms**
BP‐CVDPM: Blood Pressure Control–Cardiovascular Disease Policy Model
NHANES: National Health and Nutrition Examination Survey
JNC 7: Seventh Report of the Joint National Committee on Prevention, Detection, Evaluation, and Treatment of High Blood Pressure
SPRINT: Systolic Blood Pressure Intervention Trial
TBC: team‐based care
UI: uncertainty interval

**Clinical Perspective**

**What Is New?**

- The SPRINT (Systolic Blood Pressure Intervention Trial) intensive blood pressure goals are not readily used in practice, and head‐to‐head comparisons with other hypertension management strategies do not exist.
- A computer simulation model compared usual hypertension care targeting <140/90 mm Hg with the SPRINT protocol targeting <120/90 mm Hg, usual care targeting <130/80 mm Hg, and a pragmatic team‐based care approach targeting <130/80 mm Hg.
- In the 18.1 million SPRINT‐eligible US adults, the SPRINT intensive protocol and team‐based care were projected to reduce total cardiovascular disease events by 138 100 and 89 100 per year, respectively, and increase treatment‐related serious adverse events by 77 600 and 27 200, respectively.

**What Are the Clinical Implications?**

- Blood pressure control has worsened in the United States in recent years, and primary care practices are motivated to improve hypertension management.
- In individuals meeting the SPRINT eligibility criteria, treatment according to the SPRINT protocol is projected to prevent the most cardiovascular disease events, but adding team‐based care to usual care could be an effective strategy that partially offsets the increased risk of treatment‐related serious adverse events.
- Hypertension policy makers may consider team‐based care a pragmatic and safe approach to hypertension management that provides patients with frequent monitoring and engagement while reducing the burden on primary care providers.

In SPRINT (Systolic Blood Pressure Intervention Trial), an intensive systolic blood pressure (SBP) goal of <120 mm Hg (SPRINT intensive) compared with a standard goal of <140 mm Hg significantly reduced cardiovascular disease (CVD) events (hazard ratio [HR], 0.75; *P*<0.001) and all‐cause death (HR, 0.73; *P*=0.003) but increased the risk of treatment‐related serious adverse events (SAEs) (HR, 1.88; *P*<0.001) in adults with hypertension at high CVD risk.^1^ Compared with a standard SBP goal, the SPRINT intensive SBP goal has been estimated to be cost effective.^2^, ^3^ There are ≈18.1 million US adults that would meet SPRINT eligibility criteria, and effectively implementing intensive SBP goals could prevent a substantial number of CVD events and deaths.^4^, ^5^

However, due in part to concerns about implementing the intensive treatment protocol, the ability to replicate BP measurement in clinical practice, and the risk of SAEs, hypertension management according to the SPRINT protocol and targeting intensive SBP goals has not been readily adopted in the United States.^6^ Additionally, as individuals at the highest risk of CVD may be those most likely to experience SAEs, alternative hypertension management strategies to the intensive protocol and SBP goals of SPRINT may need to be considered in SPRINT‐eligible US adults.^7^, ^8^

Targeting a less intensive blood pressure (BP) goal, such as the 2017 Hypertension Clinical Practice Guideline (American College of Cardiology/American Heart Association [ACC/AHA])‐recommended <130/80 mm Hg, as part of usual care could be one strategy.^9^ However, no studies have evaluated the effectiveness of alternate BP goals and care management strategies in SPRINT‐eligible individuals. Additionally, team‐based care (TBC), that is, ≥2 health care providers working toward a shared clinical goal, is an effective strategy to lower BP and may help to prevent or address SAEs through proactive patient support with frequent follow‐up and monitoring.^10^, ^11^, ^12^ TBC in which a nonphysician team member (eg, pharmacist) can titrate antihypertensive medications is more effective and cost effective than usual care or TBC with physician medication titration.^10^, ^11^ Further, when targeting a BP goal of <130/80 mm Hg, TBC could approach the SBP achieved in SPRINT.^13^, ^14^ However, randomized clinical trial evidence comparing the potential benefits and harms of different hypertension management strategies in SPRINT‐eligible US adults does not exist.

To fill this evidence gap and help guide health care policy makers implementing hypertension control strategies, we therefore used a computer simulation model of hypertension care processes and CVD events to compare usual care with intensive treatment according to the SPRINT protocol, and usual care and TBC targeting the 2017 ACC/AHA BP goal. We projected the SBP, CVD, SAE, and survival outcomes over 10 years and remaining lifetime of SPRINT‐eligible US adults.

## Methods

The simulation model used for this analysis is available to interested researchers upon reasonable request and approval by the modeling team. Interested researchers must submit a research proposal and collaboration plan to Dr Bellows and sign a Creative Commons agreement. This study was approved by the Columbia University Institutional Review Board, and all participants provided written informed consent from each participating institution (Data S1). A summary of SPRINT is reported in Data S1.

### Model Overview

The current analysis used the Blood Pressure Control–Cardiovascular Disease Policy Model (BP‐CVDPM), a validated discrete event simulation of hypertension management and CVD outcomes (Figure S1).^10^, ^15^, ^16^, ^17^ The BP‐CVDPM predicts long‐term BP outcomes by simulating hypertension care processes, including visit frequency, BP measurement accuracy, probability of antihypertensive medication intensification when BP is uncontrolled, and patient medication adherence. Fatal and nonfatal CVD events (ie, coronary heart disease, stroke, and heart failure), treatment‐related intolerable adverse events and SAEs, survival, and health‐adjusted survival are projected by the model accounting for individual characteristics and hypertension treatment. The model is calibrated to reproduce contemporary rates of CVD, acute and chronic CVD death, and non‐CVD death in the United States by age and sex.^10^, ^16^, ^17^ Time progresses from one event to the next in the model (eg, office visit, SAE, CVD), and when an event occurs, the model determines the associated health outcomes, updates the individual's characteristics, and calculates the time to the next event.

### Simulated Population

The model was populated with participants from the National Health and Nutrition Examination Survey (NHANES; 1999–2018 cycles) who previously had lifetime trajectories (age 18–99 years) developed to estimate changes in CVD risk factors over their life course (ie, smoking status, SBP, diastolic BP, body mass index, diabetes, high‐density lipoprotein cholesterol, low‐density lipoprotein cholesterol, and estimated glomerular filtration rate).^10^, ^16^, ^17^, ^18^, ^19^ Of these, 2094 individuals met the SPRINT eligibility criteria (Data S2), comparable with other NHANES‐based projections from SPRINT.^4^, ^5^ To this SPRINT‐eligible NHANES sample, 2 weighting schemes were applied: (1) weighted to create a cohort resembling the baseline characteristics of SPRINT participants (SPRINT‐representative) and (2) weighted to be nationally representative of the population of SPRINT‐eligible US adults using the survey weights from NHANES (Data S2, Table S1, and Figure S2). Standardized mean differences were used to compare the published baseline characteristics of SPRINT participants with the simulated population under both weighting schemes. To ensure stable outcome estimates, all analyses sampled 25 000 individuals with replacement.

### Simulated Comparators and Hypertension Care Processes

**Figure 1:**
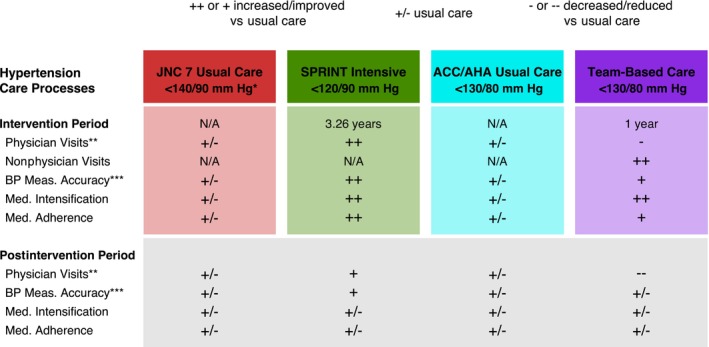
Hypertension management strategies and effects on care processes. *JNC 7 BP goal was <140/90 mm Hg for most patients and <130/80 mm Hg for patients with chronic kidney disease or diabetes if developed during the simulation. **Physician visits during the intervention period for SPRINT were according to the SPRINT protocol, that is, once per month for 3 months, then once every 3 months. For usual care, physician visits were dependent on BP control at the visit and other patient and visit characteristics (Data S3 and Table S3). During the postintervention period, increased or decreased physician visit frequency vs usual care were a result of BP control achieved during the intervention period. ***BP measurement accuracy was dependent on the number of BP readings per visit and total number of visits. In the postintervention period, increased BP measurement accuracy vs usual care was a result of a higher total number of visits. The figure shows the 4 hypertensive management strategies simulated, including the targeted BP goal, duration of the interventions, and relative effect on hypertension care processes vs usual care. ++ and + indicate strong and moderate effects, respectively, increasing or improving the process of care (eg, frequent physician visits, reduced BP measurement error), +/‐ indicates equivalence with usual care, and – and ‐ indicate strong and moderate effects, respectively, decreasing or reducing the process of care. ACC indicates American College of Cardiology; AHA, American Heart Association; BP, blood pressure; JNC 7, Seventh Report of the Joint National Committee on Prevention, Detection, Evaluation, and Treatment of High Blood Pressure; Meas., measurement; Med., medication; N/A, not applicable; and SPRINT, Systolic Blood Pressure Intervention Trial.

Individuals started the simulation meeting the SPRINT eligibility criteria and presenting at a physician office visit. The model then compared initiation of 4 hypertension management strategies (Figure 1, Table S2). In the first strategy, the Seventh Report of the Joint National Committee on Prevention, Detection, Evaluation, and Treatment of High Blood Pressure (JNC 7) usual care, individuals were assumed to receive usual care (ie, hypertension care processes were derived from national sources and published literature) and treated to BP <140/90 mm Hg or<130/80 mm Hg with chronic kidney disease or diabetes, if it developed during the simulation (Data S3 and Tables S2 and S3).^10^, ^16^, ^17^, ^20^ In the second strategy, SPRINT intensive, individuals were simulated to receive SPRINT protocol‐based hypertension care with 1 physician visit per month for 3 months followed by 1 visit every 3 months for 3.26 years (ie, median SPRINT follow‐up), and treated to the SPRINT intensive (<120/90 mm Hg) BP goal. Hypertension care processes were updated to reflect, where possible, that observed in SPRINT; others were based on published literature and calibrated as needed (Data S3).^1^, ^21^ After 3.26 years, individuals were assumed to receive usual care (ie, hypertension care processes reverted to usual care) but maintain the SPRINT BP goal. In the third strategy, ACC/AHA usual care, individuals received usual care hypertension care processes and were treated to the 2017 ACC/AHA BP goal of <130/80 mm Hg. In the fourth strategy, TBC, individuals had TBC added to usual care for 1 year and were treated to a BP goal of <130/80 mm Hg. Based on a published meta‐analysis, individuals had TBC visits with a nonphysician team member who could titrate medications (eg, pharmacist) once every 6 weeks during the first year, and other hypertension care processes from prior analyses of TBC.^10^, ^17^ After the first year of TBC, individuals received usual care alone and maintained a BP goal <130/80 mm Hg.

### Outcomes

Primary outcomes were SBP, fatal or nonfatal CVD events, CVD‐related death, and fatal or nonfatal SAEs. SAEs considered possibly or definitely related to treatment were as defined in SPRINT as follows: “fatal or life threatening, resulting in significant or persistent disability,” or “requiring or prolonging hospitalization.”^1^, ^2^, ^10^, ^16^, ^17^, ^22^ It was assumed in the model that all SAEs would result in hospitalization. Secondary outcomes included all‐cause death, life years (undiscounted), healthy life years (undiscounted), and number of physician and nonphysician visits. Healthy life years quantifies the number of years a population can anticipate living in good health and can be simulated by adjusting life years for years lived with disease and years lost due to premature death.^23^ We used estimates from published literature to adjust life years for acute and chronic CVD, intolerable adverse events, SAEs, hypertension, dyslipidemia, obesity, diabetes, end‐stage renal disease, and other comorbidities with aging (Table S3).^2^, ^24^, ^25^, ^26^, ^27^, ^28^

### Model Inputs

The risk of incident CVD events and non‐CVD death were from Cox proportional hazards models derived in the National Heart, Lung, and Blood Institute Pooled Cohorts Study and account for age, race, smoking status, SBP, body mass index, diabetes, high‐density lipoprotein cholesterol, low‐density lipoprotein cholesterol, and estimated glomerular filtration rate (Table S3).^19^, ^29^, ^30^ Age‐ and sex‐specific secondary or recurrent CVD event rates and CVD death were derived from the Centers for Disease Control and Prevention, National Inpatient Sample, and National Vital Statistics System, and the dynamic population version of the CVD Policy Model.^10^, ^16^, ^17^ Reductions in risk of CVD events per 10 mm Hg SBP lowering were derived from a published meta‐analysis.^31^ The risk of treatment‐related intolerable adverse events and SAEs was dependent on the number of antihypertensive medications used and derived from published literature.^1^, ^10^, ^16^, ^17^, ^22^, ^32^, ^33^ The probability that an SAE was fatal was dependent on age and derived from the National Inpatient Sample.^2^, ^10^, ^16^, ^17^ Other model inputs were synthesized from published literature and public data sources.^1^, ^2^, ^15^, ^22^, ^24^, ^32^, ^33^, ^34^, ^35^, ^36^, ^37^, ^38^, ^39^, ^40^, ^41^, ^42^

### Model Calibration

The BP‐CVDPM was calibrated in the SPRINT‐representative cohort to reproduce the SBP achieved, number of antihypertensive medications used, and CVD events observed over the median follow up in SPRINT (Data S4, Table S4, and Figure S3). Additional calibration was not needed to reproduce the rates of CVD‐related death and SAEs considered possibly or definitely related to treatment. To ensure that the model reproduced SBP reductions with TBC versus usual care at 1 year, the model projections were compared with a published meta‐analysis (Table S5).^10^ To balance computational efficiency with capturing uncertainty in model input sampling, the model calibration was tested by running 200 probabilistic iterations in which model parameters were randomly sampled from prespecified statistical distributions. The mean of the rates and 95% uncertainty intervals (UIs; 2.5th to 97.5th percentile of means) from the 200 iterations were calculated, as well as the proportion of iterations that resulted in a rate within the 95%CI reported in SPRINT.

### Statistical Analysis

The BP‐CVDPM was constructed in TreeAge Pro 2021 (TreeAge Software, LLC, Williamstown, MA) and other analyses were performed in R version 4.2.1 (R Foundation for Statistical Computing, Vienna, Austria) and Microsoft Excel (Microsoft Corporation, Redmond, WA). In the SPRINT‐representative population weighted to resemble SPRINT participants, a 10‐year time horizon was used. When weighted to resemble SPRINT‐eligible US adults, both 10‐year and remaining lifetime (age 100 years or death) time horizons were used and outcomes were scaled up to the 18.1 million SPRINT‐eligible US adults (Data S5).^4^ The means and 95% UI are reported for all outcomes of the main analyses from running 200 probabilistic model iterations, the same as the approach used for calibration testing.

### Sensitivity and Scenario Analyses

One‐way sensitivity analyses varied hypertension care process parameters and duration of the SPRINT and TBC interventions across a range of plausible values while holding all other model parameters constant in the population weighted to resemble SPRINT participants. In the primary analyses, it was assumed that SBP lowering was not associated with a reduction in non‐CVD death. However, a reduction in all‐cause death with intensive versus standard SBP goals not fully explained by the reduction in CVD‐related deaths was observed in SPRINT.^1^ Therefore, a scenario analysis was performed in which a reduction in risk of non‐CVD death per 10 mm Hg SBP lowering was included and the model calibrated to replicate the all‐cause death results observed in SPRINT. An additional scenario analysis assumed usual care hypertension processes throughout the entire time horizon but targeted the BP goals for SPRINT intensive.

## Results

### Primary Analysis

#### Impact of Hypertension Management in SPRINT‐Representative Cohort

**Figure 2:**
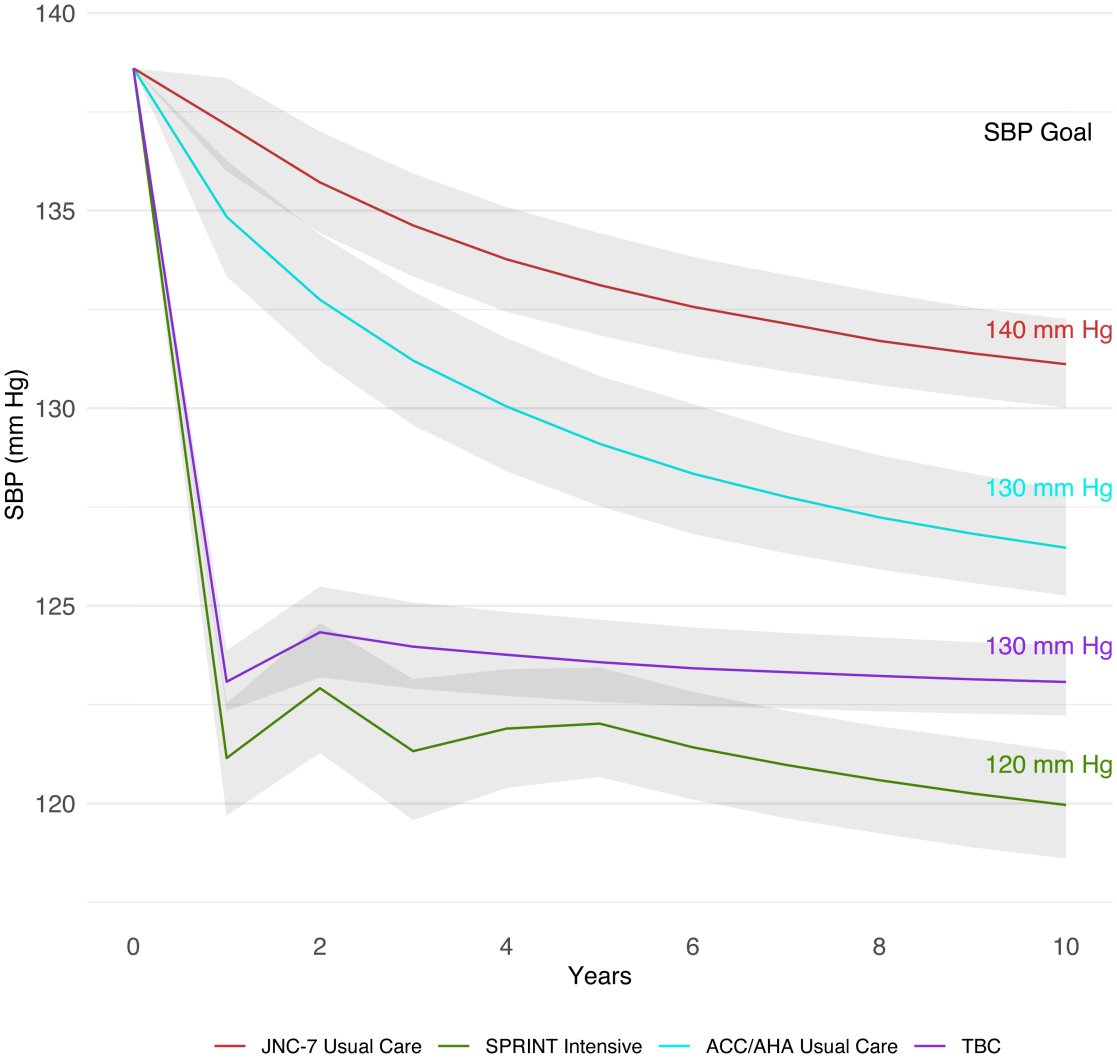
Projected mean systolic blood pressured achieved. The figure shows the projected mean SBP achieved each year over 10 years with each hypertension management strategy in a population weighted to resemble SPRINT. The results are presented as the mean and 95% uncertainty interval (gray shaded regions) calculated by running 200 probabilistic iterations in which the model was run repeatedly when randomly sampling input parameters from prespecified statistical distributions. ACC indicates American College of Cardiology; AHA, American Heart Association; JNC 7, Seventh Report of the Joint National Committee on Prevention, Detection, Evaluation, and Treatment of High Blood Pressure; SBP, systolic blood pressure; SPRINT, Systolic Blood Pressure Intervention Trial; and TBC, team‐based care.

At 10 years in the SPRINT‐representative cohort, the mean SBP was projected to decrease from 138.6 mm Hg at baseline to 131.3 (95% UI, 130.2–132.5) mm Hg with JNC 7 usual care, 120.2 (95% UI, 118.9–121.5) mm Hg with SPRINT intensive, 126.9 (95% UI, 125.7–128.4) mm Hg with ACC/AHA usual care, and 123.5 (95% UI, 122.6–124.5) mm Hg with TBC (Figure 2). Additionally, the BP goal, which varied for each strategy, was projected to be achieved by 80.3% (95% UI, 74.5%–85.5%) of individuals with JNC 7 usual care (<140/90 mm Hg or <130/80 mm Hg with chronic kidney disease or diabetes), 56.4% (95% UI, 48.1%–65.7%) with SPRINT intensive (<120/90 mm Hg), 73.5% (95% UI, 65.7%–79.8%) with ACC/AHA usual care (<130/80 mm Hg), and 87.6% (95% UI, 82.8%–91.5%) with TBC (<130/80 mm Hg). Relative to JNC 7 usual care at 10 years, 61 (95% UI, 54–68) CVD events per 1000 individuals were projected to be prevented with SPRINT intensive, 17 (95% UI, 12–22) with ACC/AHA usual care, and 48 (95% UI, 43–55) with TBC (Table 1).

**Table 1:** Projected Events per 1000 Individuals in SPRINT‐Representative US Adults at 10 Years

Outcome	JNC 7 usual care	SPRINT intensive	ACC/AHA usual care	Team‐based care
CVD events	247 (227–273)	187 (163–214)	231 (206–256)	199 (177–228)
Coronary heart disease	136 (119–155)	109 (91–132)	129 (112–149)	112 (94–134)
Stroke	55 (47–61)	35 (27–41)	49 (41–55)	40 (33–46)
Heart failure	57 (47–67)	42 (33–51)	53 (43–62)	47 (37–58)
SAEs	42 (34–50)	77 (61–93)	55 (44–66)	49 (40–59)
Death from any cause	179 (163–196)	168 (147–190)	176 (158–195)	168 (151–189)
CVD‐related deaths	53 (47–58)	41 (36–47)	49 (44–54)	43 (38–49)
SAE‐related deaths	1 (0–2)	1 (1–2)	1 (1–2)	1 (0–1)

The table shows the projected number of events per 1000 individuals over 10 years with each hypertension management strategy in a population weighted to resemble SPRINT participants. The results are presented as the mean and 95% uncertainty interval calculated by running 200 probabilistic iterations in which the model was run repeatedly when randomly sampling input parameters from prespecified statistical distributions. ACC indicates American College of Cardiology; AHA, American Heart Association; CVD, cardiovascular disease; JNC 7, Seventh Report of the Joint National Committee on Prevention, Detection, Evaluation, and Treatment of High Blood Pressure; SPRINT, Systolic Blood Pressure Intervention Trial; and SAE, serious adverse event.

Compared with JNC 7 usual care, the number of SAEs per 1000 individuals was estimated to increase by 35 (95% UI, 24–45) with SPRINT intensive, 13 (95% UI, 10–16) with ACC/AHA usual care, and 6 (95% UI, 0–12) with TBC. SPRINT intensive and TBC were estimated to have a comparable incidence of SAEs for the first 2 years, but fewer treatment intensifications with TBC over the remaining time horizon due to a greater proportion reaching the BP target resulted in fewer SAEs with TBC (Figure S4). Compared with JNC 7 usual care, 12 (95% UI, 10–14) CVD‐related deaths per 1000 individuals were projected to be prevented with SPRINT intensive, 4 (95% UI, 3–6) with ACC/AHA usual care, and 10 (95% UI, 8–12) with TBC. SPRINT intensive was projected to have the most physician visits over 10 years and TBC the fewest (Table S6).

#### National Impact of Hypertension Management in SPRINT‐Eligible US Adults

**Figure 3:**
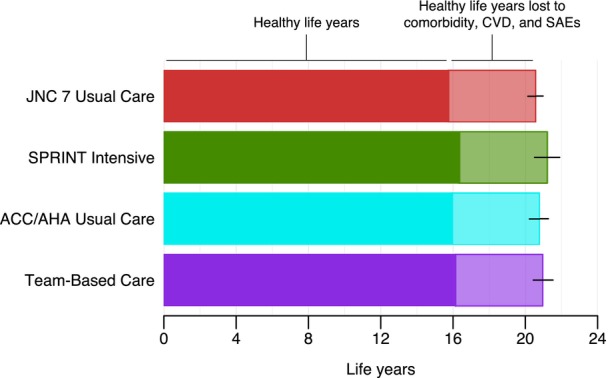
Estimated survival and healthy life years in US adults meeting SPRINT eligibility criteria. The figure shows the mean survival from baseline with each hypertension management strategy in a population weighted to all 18.1 million SPRINT‐eligible US adults. The results are presented as the mean and 95% uncertainty interval (error bar) calculated by running 200 probabilistic iterations in which the model was run repeatedly when randomly sampling input parameters from prespecified statistical distributions. The solid region indicates the healthy life years, which adjusted survival for acute CVD events, chronic CVD, intolerable adverse events, SAEs, hypertension, dyslipidemia, obesity, diabetes, end‐stage renal disease, and other comorbidities with aging. ACC indicates American College of Cardiology; AHA, American Heart Association; CVD, cardiovascular disease; JNC 7, Seventh Report of the Joint National Committee on Prevention, Detection, Evaluation, and Treatment of High Blood Pressure; SAE, serious adverse event; and SPRINT, Systolic Blood Pressure Intervention Trial.

When weighted to represent the 18.1 million SPRINT‐eligible US adults, SBP was projected to decrease from 138.7 mm Hg at baseline to 133.8 (95% UI, 132.9–134.7) mm Hg with JNC 7 usual care, 121.4 (95% UI, 119.9–122.9) mm Hg with SPRINT intensive, 129.1 (95% UI, 127.8–130.5) mm Hg with ACC/AHA usual care, and 126.2 (95% UI: 125.3–127.0) mm Hg with TBC at 10 years. Relative to JNC 7 usual care, an estimated 138 100 (95% UI, 117 800–159 900) total CVD events could be prevented per year with SPRINT intensive, 33 900 (95% UI, 19 800–49 400) with ACC/AHA usual care, and 89 100 (95% UI, 72800–104 600) with TBC (Table 2). An estimated 117 100 CVD‐related deaths per year (≈0.6% of all SPRINT‐eligible US adults) were estimated to occur with JNC 7 usual care. Compared with JNC 7 usual care, an estimated 25 900 (95% UI, 20900–31 800) CVD‐related deaths could be prevented per year with SPRINT intensive, 5800 (95% UI, 300–10 400) with ACC/AHA usual care, and 17 300 (95% UI, 10 400–23 200) with TBC. However, SPRINT intensive was projected to increase the number of SAEs per year by 77 600 (95% UI, 59 900–92 500), ACC/AHA usual care by 33 300 (95% UI, 25 500–41 900), and TBC by 27 200 (95% UI, 19 500–34 700) compared with JNC 7 usual care. When simulating the remaining lifetime (up to age 100 years or death), healthy life years were estimated to increase versus JNC 7 usual care by 0.6 (95% UI, 0.3–0.8) years with SPRINT intensive, 0.2 (95% UI: 0.1–0.3) years with ACC/AHA usual care, and 0.4 (95% UI, 0.2–0.5) years with TBC (Figure 3).

**Table 2:** Projected Events per Year in US Adults Meeting SPRINT Eligibility Criteria

Outcome	JNC 7 usual care	SPRINT intensive	ACC/AHA usual care	Team‐based care
CVD events	603 800 (576 700–630 000)	465 700 (428 300–512 100)	570 000 (533 300–600 900)	514 700 (476 200–552 400)
Coronary heart disease	309 300 (292 100–329 200)	256 100 (227 100–291 500)	297 000 (276 800–322 500)	276 100 (253 600–304 300)
Stroke	153 300 (146 900–160 600)	101 400 (88 300–113 000)	137 600 (125 300–149 100)	118 100 (104 900–128 900)
Heart failure	141 200 (127 700–154 100)	108 200 (91 700–123 900)	135 400 (120 800–150 300)	120 500 (105 300–137 200)
SAEs	58 000 (43 200–71 500)	135 600 (104 800–161 800)	91 400 (70 300–114 700)	85 300 (66 500–102 400)
Death from any cause	381 800 (361 200–401 700)	357 100 (325 900–390 100)	379 500 (353 000–404 700)	360 200 (334 100–384 700)
CVD‐related death	117 100 (111 600–121 600)	91 200 (83 900–98 800)	111 300 (103 300–118 800)	99 800 (92 000–108 600)
SAE‐related deaths	900 (400–1600)	2400 (1100–4100)	1500 (800–2500)	1500 (700–2600)

The table shows the projected number of events per year with each hypertension management strategy in the 18.1 million US adults who would meet the SPRINT eligibility criteria. The results are presented as the mean and 95% uncertainty interval calculated by running 200 probabilistic iterations in which the model was run repeatedly when randomly sampling input parameters from prespecified statistical distributions. ACC indicates American College of Cardiology; AHA, American Heart Association; CVD, cardiovascular disease; JNC 7, Seventh Report of the Joint National Committee on Prevention, Detection, Evaluation, and Treatment of High Blood Pressure; SPRINT, Systolic Blood Pressure Intervention Trial; and SAE, serious adverse event.

### Sensitivity and Scenario Analyses

The effects of independently varying each hypertension process parameter and the duration of the SPRINT and TBC intervention on mean SBP achieved and CVD events, CVD‐related deaths, and SAEs per 1000 individuals over 10 years in the SPRINT‐representative cohort are shown in Figure S5. When a relative risk per 10‐mm Hg SBP lowering on non‐CVD death was included, the deaths from any cause prevented per year was estimated to increase to 118 600 with SPRINT intensive and 87 300 with TBC compared with JNC 7 usual care in SPRINT‐eligible US adults, and healthy life years were estimated to increase by 2.1 and 1.4 years, respectively (Table S7). The results when simulating hypertension management with usual care throughout the entire time horizon when treating to the SPRINT intensive BP goals are shown in Table S8.

## Discussion

### Summary of Findings

As BP control has declined in the United States in recent years, health systems must prioritize hypertension management and invest in effective strategies. In this analysis, we used a validated computer simulation model to project and compare the effectiveness of different hypertension management strategies, including JNC 7 usual care, SPRINT intensive, ACC/AHA usual care, and TBC, over 10 years and expected remaining lifetime in US adults who would meet the SPRINT eligibility criteria. Though intensive treatment according to the SPRINT protocol targeting an intensive SBP goal was estimated to be the most effective in reducing SBP, preventing CVD events, and increasing survival and healthy life years, it was also projected to result in the greatest number of SAEs. Treatment with 1 year of TBC targeting the 2017 ACC/AHA BP goal was estimated to be the second most effective strategy in reducing SBP, preventing CVD events, and increasing survival and healthy life years while resulting in a substantially lower number of SAEs compared with SPRINT intensive. The intensive SPRINT protocol and SBP goal of <120 mm Hg has not been readily adopted in the United States, due in large part to clinical inertia surrounding the ability to replicate BP measurement in clinical practice and concerns about SAEs. A pragmatic approach may be to consider adding TBC to usual care with a less intensive BP goal, such as the 2017 Hypertension Clinical Practice Guidelines recommended <130/80 mm Hg, which could substantially reduce morbidity and death in a SPRINT‐eligible population at high risk of CVD.

### Comparison With Previous Literature

Prior publications have estimated the number of CVD events and deaths that could be prevented each year if SPRINT‐eligible US adults were to receive SPRINT intensive treatment.^4^, ^5^ Our analysis adds to these by examining longer time horizons of 10 years and remaining lifetime (versus ≤5 years), considering total CVD events (versus first CVD event during SPRINT follow‐up), and examining a return to the usual care process of hypertension management while continuing to target the BP goals from SPRINT (versus treatment according to SPRINT protocol). Additionally, the BP‐CVDPM allowed us to project and compare treatment according to the SPRINT protocol with other hypertension management strategies and BP goals, including modifications to the SPRINT protocol; to our knowledge, this is the first study to address this gap. The survival estimate with SPRINT intensive in our analysis (ie, 21.2 years) is similar to previously published estimates derived from simulation models, cohort data, and actuarial methods (ranging from 19.0 to 24.5 years) but is longer than another from a simulation model (14.3 years).^2^, ^3^, ^43^, ^44^

### Implications for Clinical Practice

Similar to other publications, the current study projected that SPRINT intensive was likely to be the most effective strategy for reducing CVD morbidity and death in SPRINT‐eligible adults. However, in some populations, for example, older adults with multimorbidity or frailty, the risk of SAEs may be an important treatment consideration. Though analysis of SPRINT participants aged ≥75 years did not find a significantly increased risk of SAEs with intensive versus standard SBP goals and most adverse events were transient, concerns have been raised of the representativeness of SPRINT of the general population and the lack of trial evidence to support treatment decisions in older adults with multimorbidity or frailty.^45^, ^46^, ^47^, ^48^ Additionally, though most individuals in SPRINT were treated at baseline, observational data show a significant increase in the absolute risk of SAEs with antihypertensive treatment initiation in older adults and those with moderate to severe frailty.^49^ In this context, our analysis provides a framework to consider approaches that weigh the CVD benefits of an intensive SBP goal with concerns about SAEs. Only about 56% of individuals in the SPRINT intensive arm were projected to achieve the intensive BP goal of <120/90 mm Hg at 10 years; this resulted in new medication intensifications and higher rates of SAEs over time. Conversely, individuals in the TBC arm were projected to rapidly achieve similar SBP lowering but with about 88% achieving the BP goal of <130/80 mm Hg at 10 years; this resulted in fewer subsequent medication changes and, long term, fewer SAEs. However, estimating an individual patient's risk of experiencing an SAE remains difficult. Shared decision making can help patients and providers determine BP goals (eg, consider other guideline recommendations such as JNC 8) and weigh the risks of SAEs with the potential benefits of initiating or intensifying antihypertensive medication regimens. Clinicians and health systems designing hypertension programs should consider both the short‐ and long‐term potential risk for SAEs with intensive SBP goals, particularly as the population ages and individuals develop multimorbidity and frailty. One year of TBC added to usual care targeting the 2017 ACC/AHA BP goal may be a pragmatic approach that balances SBP lowering and reduction in CVD risk with frequent monitoring and patient engagement and a long‐term reduction in SAEs. Further research comparing TBC targeting the ACC/AHA BP goal with current usual care, eg, pragmatic clinical trial, may be helpful to shift clinical practice and reimbursement incentives and speed adoption.

### Strengths and Limitations

Our analysis is strengthened by using the BP‐CVDPM, which provides a flexible approach to simulating the processes of hypertension care and how changes to management might affect outcomes. This allows comparisons of hypertension management strategies for which there are no short‐ or long‐term head‐to‐head trial comparisons. However, the long‐term projections are based on assumptions about the model inputs and patient and provider behaviors in clinical practice. Pragmatic clinical trials may be needed to confirm the effectiveness of TBC in SPRINT‐eligible patients projected in our analysis. The model used a traditional *start low and go slow* antihypertensive medication regimen that started with lower doses that were titrated upward before adding subsequent medications. Antihypertensive medication regimens vary across health care systems (eg, adoption of treatment algorithms with early use of fixed‐dose combination regimens) and from provider to provider, which also likely differ from the SPRINT treatment formulary. Similarly, the model does not consider class or regimen specific effects, such as risks of hypokalemia or hypomagnesemia with thiazide diuretics versus risks of hyperkalemia with angiotensin‐converting enzyme inhibitors and angiotensin II receptor blockers medications or angioedema with angiotensin‐converting enzyme inhibitors. Instead, the model used the average BP‐lowering effects and risks of treatment‐related adverse events derived from large meta‐analyses. The model also assumed that treatment‐related SAEs resulted in a hospitalization and therefore may not capture SAEs and fatal SAEs that occur out of the hospital. However, the model reproduced the mean SBP, CVD, and SAE outcomes observed in SPRINT and has previously replicated outcomes from trials and observational data.^10^, ^15^, ^16^, ^17^ Though the number of individuals from our simulated population of NHANES participants meeting SPRINT eligibility criteria was comparable to other studies, a larger sample size may have provided more precise estimates. However, the sample was limited by the ability to match to SPRINT eligibility criteria.

## Conclusions

As BP control has declined in the United States in recent years, health systems must prioritize hypertension management and invest in effective strategies. Compared with usual care hypertension management targeting the JNC 7 BP goals, the SPRINT intensive protocol and BP goal (<120/90 mm Hg) and TBC added to usual care targeting the 2017 ACC/AHA BP goal (<130/80 mm Hg) are projected to reduce SBP, CVD events, and CVD‐related deaths and increase healthy life years in US adults meeting the SPRINT eligibility criteria. However, TBC added to usual care is projected to substantially reduce the long‐term number of SAEs compared with the intensive SPRINT treatment protocol. Health care policy makers may consider adoption of TBC with nonphysician providers who can titrate antihypertensive medications and provide patients with frequent monitoring and engagement as a pragmatic approach to reduce the burden of CVD in this high‐risk population.

## Sources of Funding

This analysis was funded by R01HL130500 (Dr Moran) and K01HL140170 (Dr Bellows) from the National Heart, Lung, and Blood Institute (NHLBI; Bethesda, MD). Dr Cohen was supported by R01HL157108 and R01HL153646 from the NHLBI (Bethesda, MD) and R01AG074989 from the National Institute on Aging (Bethesda, MD). Dr Bress was supported by R01AG074989, R01AG0658505, and K24AG080168 from the National Institute on Aging (Bethesda, MD). Dr Zhang was supported by R01HL158790 from the NHLBI (Bethesda, MD). Dr Kronish was supported by R01HL152699 from the NHLBI (Bethesda, MD), and Dr King was supported by R01HL157439 from the NHLBI (Bethesda, MD). Dr Sheppard was supported by funding from the Wellcome Trust/Royal Society via a Sir Henry Dale Fellowship (ref: 211182/Z/18/Z). This research was funded in part by the Wellcome Trust (211 182/Z/18/Z). For the purpose of open access, the author has applied a CC BY public copyright license to any author accepted manuscript version arising from this submission. The funders had no role in the design and conduct of the study; collection, management, analysis, and interpretation of the data; preparation, review, approval of the manuscript; or decision to submit for publication.

## Disclosures

None.

## Supporting information

Data S1–S5
Tables S1–S8
Figures S1–S5
References^50^, ^51^
