# Supplementary material for: Effectiveness of Hypertension Management Strategies in SPRINT‐Eligible US Adults: A Simulation Study
Source: J Am Heart Assoc. 2024 Jan 12;13(2):e032370. doi: 10.1161/JAHA.123.032370 (PMC10926802; doi:10.1161/JAHA.123.032370)
Supplement: Supplementary file 1 — Data S1–S5 Tables S1–S8 Figures S1–S5 References 50 , 51 [file JAH3-13-e032370-s001.pdf]

SUPPLEMENTAL MATERIAL

SUPPLEMENTAL METHODS

Data S1. SPRINT Overview

The Systolic Blood Pressure Intervention Trial (SPRINT) included 9,361 participants aged ≥50 years with SBP 130-180 mmHg and an increased risk of cardiovascular disease (CVD) events (i.e., clinical or subclinical CVD, an estimated glomerular filtration rate [eGFR] 20-59 mL/min/1.73 m^2, Framingham 10-year CVD risk score ≥15%, or aged ≥75 years) and without diabetes, prior stroke, or prior heart failure (HF).[1] Participants were randomly assigned to treatment with an intensive (<120/90 mmHg) or a standard (<140/90 mmHg) blood pressure (BP) goal. Participants were followed monthly for the first three months and every three months thereafter for a median follow-up of 3.26 years. The primary outcome was composite CVD events, including fatal and non-fatal coronary heart disease, stroke, or HF. Secondary outcomes included the individual event types in the primary outcome, CVD-related death, all-cause mortality, and treatment-related serious adverse events (SAEs). The study protocols for SPRINT were approved by the Institutional Review Boards (IRBs) at each participating institution and all participants provided written informed consent.

Data S2. Simulated Population

A cohort of 19,860 National Health and Nutrition Examination Survey (NHANES) participants from the 1999-2000 to 2017-2018 cycles who have had lifetime CVD risk factor trajectories from participants in the National Heart, Lung, and Blood Institute (NHLBI) Pooled Cohorts Study previously developed was used to identify participants meeting SPRINT eligibility criteria.[10,16,17] The IRB at the National Center for Health Statistics of the Centers for Disease Control and Prevention (CDC) approved the NHANES protocols, and all participants provided written informed consent. The study protocols for the NHLBI cohorts were approved by the IRBs at each participating institution and all participants provided written informed consent.

Included participants were: (1) aged ≥50 years; (2) had a systolic BP (SBP) estimated to be between 130-180 mmHg without treatment and SBP <180 mmHg when using one antihypertensive medication, SBP <170 mmHg when using two antihypertensive medications, SBP <160 mmHg when using three antihypertensive medications, and SBP <150 mmHg when using four antihypertensive medications; and (3) had at least one high risk condition: history of coronary heart disease, an estimated glomerular filtration rate (eGFR) 20-59 mL/min, a 10-year risk of CVD Framingham risk score ≥15%, or were aged ≥75 years. Participants were excluded if they had a history of diabetes, stroke, or HF, or had an eGFR <20 mL/min.

A calibrated propensity score-based approach was used to weight NHANES participants to resemble the baseline characteristics of SPRINT participants. First, SPRINT-eligible NHANES participants were combined with a hypothetical cohort matching the baseline characteristics of SPRINT participants. Then, a propensity score model was developed (R “twang” package) to generate new weights for SPRINT-eligible NHANES participants to match the SPRINT baseline characteristics. Then, to improve the replication of the baseline characteristics of SPRINT participants, the new weights were calibrated (R “survey” package) using categorical variables and quantiles of continuous variables. Finally, the calibrated weights were truncated at the 1st and 99th percentile to prevent extreme weights. Analyses were run using two weighting schemes: (1) when weighted to resemble the baseline characteristics of SPRINT participants and (2) when weighted to be nationally representative of SPRINT-eligible US adults using the survey weights from NHANES. Characteristics of the simulated population were compared with the published baseline characteristics of SPRINT participants using standardized mean differences (SMD).

Compared with published baseline characteristics of SPRINT participants, the simulated population of SPRINT-eligible NHANES participants weighted to resemble the baseline characteristics of SPRINT participants had an SMD <0.1 for most characteristics, a threshold often used to determine if covariate balance has been achieved (**Table S2**).[1] The simulated population had baseline SBP similar to that observed in SPRINT participants (**Figure S2**).[1,43] Diastolic BP (SMD = 0.112), eGFR (SMD = 0.165), total cholesterol (SMD = 0.106), and Framingham risk score (SMD = 0.145) remained above this threshold. However, the proportion meeting the increased CVD risk criteria due to chronic kidney disease (SMD = 0.040) and a Framingham risk score ≥15% (SMD = 0.048) were similar. When using the NHANES survey weights, SPRINT-eligible NHANES participants had an SMD ≥0.1 for most characteristics when compared with SPRINT participants.

Data S3. Hypertension Care Processes

Antihypertensive Medication Regimen

As in prior analyses using the BP-CVD Policy Model (BP-CVDPM), specific medication classes were not simulated, and the average BP lowering effects by defined dose were derived from large meta-analyses.[10,16,17,32,33] In the current study, the antihypertensive medication dose definitions (quarter-standard, half-standard, and standard) and average effects were updated to better reflect current clinical practice and align with the 2017 American College of Cardiology/American Heart Association (ACC/AHA) BP guideline.[9,32,33] The expected average BP reductions with antihypertensive treatment are shown in **Table S3**.

Antihypertensive medication titration schedule was similar to the approach used and validated in prior analyses.[10,15-17,50] When an individual's baseline SBP before treatment is <20 mmHg above goal, treatment begins monotherapy at a quarter-standard dose. When upward titration is needed, monotherapy is intensified to a half-standard dose and then a standard dose.

Subsequently, additional medications are added and titrated in the same manner. When an individual's baseline SBP before treatment is ≥20 mmHg above goal, treatment initiates with monotherapy at a standard dose. When upward titration is needed, a second standard dose medication is added and then a third standard dose medication. Subsequent medications are added at a quarter-standard dose and titrated upward. The maximum number of medication classes that an individual can receive is five. As antihypertensive medication doses are not available in NHANES, it was assumed that individuals using medications would be on a half-standard dose of the last medication added. For example, an individual using two antihypertensive medications at baseline was assumed to be on one standard dose plus one half-standard dose medication.

Usual Care Office Visit Frequency

The time between usual care physician visits when BP was controlled was stratified by age and derived from the mean number of hypertension-related office visits per year from the Medical Expenditure Panel Survey (MEPS) available from the Agency for Healthcare Research and Quality (**Table S3**). As in prior analyses, the time between physician visits with an uncontrolled BP was derived from published literature and adjusted for individual and visit characteristics (e.g., increased SBP and DBP since last visit, age, and treatment intensification all reduced the number of weeks until the next visit).[10,15-17,34]

BP Measurement Accuracy

The accuracy of BP measurements was derived from published literature and stratified by the number of office visits and number of BP measurements taken per office visit.[51] At each office visit, the BP-CVDPM randomly samples BP measurement error as the difference between underlying and measured BP.[10,15-17,50] The BP measurement error is inversely related to the number of office visits and number of BP measurements taken per office visit (**Table S3**). In

SPRINT, BP was measured using automated office BP monitoring and the average of three readings was used.[1] As no standard protocol for BP measurement exists for team-based care (TBC) visits with a non-physician team member, we assumed the same approach used in prior simulations of TBC using the BP-CVDPM, which was modeled on the Los Angeles Barbershop Blood Pressure Study.[10,13,14,17] It was assumed that TBC visit had three BP readings per visit. It was assumed that each office visit with a physician had a single BP measurement. The hypertension management replicating the SPRINT Intensive arm therefore had reduced BP measurement error for the duration of the intervention (i.e., 3.26 years) compared with usual care due to increased number of office visits and increased number of BP measures per visit. As the TBC was added to usual care for one year, a similar reduction in BP measurement error compared with usual care alone was achieved.

Data S4. Model Calibration and Validation

The BP-CVDPM was calibrated to reproduce the published outcomes from SPRINT. Calibration was conducted in stages, with the SBP and number of antihypertensive medications first replicated in both SPRINT arms by calibrating the probability of medication intensification and adherence, as in prior analyses.[10,16,17,21] Both the SBP achieved at 3.26 years and SBP changes over time were used to inform the calibration (**Table S4** and **Figure S3**). Then, the intercept of the incident CVD and non-CVD death risk equations, the distribution of incident CHD event types (myocardial infarction, cardiac arrest, or other CHD), and probability of HF after a myocardial infarction were calibrated to reproduce the published event rates (per 100 person-years) for the SPRINT standard arm. Benefit with intensive SBP goals was assumed to be achieved via the relative risks per 10 mmHg lowering from a published meta-analysis.[31]

Data S5. Scaling Up Estimates to SPRINT-Eligible US Population

To estimate the effect of scaling up each strategy to the 18.1 million US adults that meet the SPRINT eligibility criteria, outcomes were projected over 10 years when the simulated population was weighted to be nationally representative of SPRINT-eligible US adults using the survey weights from NHANES. Event rates per person-year and means over 10 years were multiplied by 18.1 million individuals. The 10-year cumulative incidence proportion was converted to a 1-year cumulative incidence proportion, assuming a constant rate, using the following formula: 1-year probability = 1 - (1 - 10-year probability)^(1 year/10 years). The 1-year probability was then multiplied by 18.1 million individuals.

Table S1. Baseline Characteristics of SPRINT Participants and SPRINT-eligible NHANES Participants.

Characteristic	SPRINT Participants	SPRINT-eligible NHANES Participants Weighted to Resemble SPRINT	SMD	SPRINT-eligible NHANES Participants	SMD
Age (year)	67.9 (9.5)	68.6 (9.2)	0.075	68.3 (9.9)	0.041
Female	35.6%	37.3%	0.035	40.1%	0.093
Race			0.066		0.643
Non-Hispanic White	57.7%	59.3%		81.0%	
Non-Hispanic Black	29.9%	27.1%		7.7%	
Hispanic	10.5%	11.4%		6.9%	
Other	1.9%	2.2%		4.3%	
SBP (mmHg)	139.7 (15.6)	138.6 (14.0)	0.074	138.7 (13.8)	0.068
DBP (mmHg)	78.1 (12.0)	76.8 (11.1)	0.112	71.6 (12.6)	0.528
Distribution of SBP			0.054		0.101
≤132 mmHg	33.5%	34.4%		34.1%	
132-145 mmHg	32.5%	34.2%		36.2%	
≥145 mmHg	34.0%	31.5%		29.6%	
eGFR (mL/min)	71.8 (20.6)	75.1 (19.5)	0.165	78.9 (16.8)	0.378
Total cholesterol (mg/dL)	190.1 (41.2)	194.6 (43.7)	0.106	207.0 (41.7)	0.408
HDL cholesterol (mg/dL)	52.9 (14.5)	53.7 (15.1)	0.054	54.0 (15.4)	0.074
Statin use	43.3%	43.2%	0.002	35.0%	0.171
Smoking status			0.062		0.134
Never	44.0%	47.5%		44.2%	
Current	13.2%	12.3%		17.7%	
Former	42.4%	40.2%		38.0%	
Framingham CVD risk score	24.8% (12.6%)	23.1% (10.7%)	0.145	21.9% (9.3%)	0.262
BMI (kg/m^2)	29.9 (5.8)	29.8 (5.7)	0.017	28.6 (5.5)	0.230
Antihypertensives agents	1.8 (1.0)	1.8 (1.0)	<0.001	0.9 (1.0)	0.841
Not using antihypertensives	9.4%	11.6%	0.072	45.0%	0.873
Increased CVD risk					
Age ≥75 years	28.2%	30.7%	0.055	28.9%	0.015
Chronic kidney disease	28.3%	26.5%	0.040	13.4%	0.373
Clinical or subclinical CVD	20.1%	20.5%	0.001	13.4%	0.180
Framingham risk score ≥15%	75.9%	73.8%	0.048	80.1%	0.116

BMI – body mass index, CVD – cardiovascular disease, DBP – diastolic blood pressure, eGFR – estimated glomerular filtration rate, HDL – high density lipoprotein, NHANES – National Health and Nutrition Examination Survey, SBP – systolic blood pressure, SMD – standardized mean differences, SPRINT - Systolic Blood Pressure Intervention Trial.

Note: The table compares the published baseline characteristics of SPRINT participants with SPRINT-eligible NHANES participants when weighted to resemble SPRINT participants and when weighted using the NHANES survey weights. Values are presented as mean (SD) or percentage. An SMD value <0.1 was used to determine if the characteristics were sufficiently similar between SPRINT participants and SPRINT-eligible NHANES participants weighted to resemble SPRINT.

Table S2. Model Key Assumptions, Inputs, and Set Up.

Model Component	Assumptions and Inputs
**Interventions**	
JNC 7 Usual Care	• BP goal: <140/90 mmHg and <130/80 mmHg with diabetes or chronic kidney disease[20]<br>• Duration of intervention: N/A<br>• Physician visit frequency: according to usual care, dependent on BP control, individual characteristics, visit characteristics[10,15-17,34]<br>• BP measurement accuracy: assumed 1 BP measure per visit[10,15-17,50]<br>• Medication intensification and adherence: according to usual care, probability of medication intensification[15] and adherence[15,35,36]
SPRINT Intensive	• BP goal: <120/90 mmHg[1]<br>• Duration of intervention: 3.26 years[1]<br>• Physician visit frequency: once every month for the first 3 months; once every 3 months starting from the 4th month until 3.26 years;[1] according to usual care after 3.26 years[10,15-17,34]<br>• BP measurement accuracy: 3 BP measures per visit and greater number of visits results in decreased measurement error relative to usual care[10,15-17,50]<br>• Medication intensification and adherence: calibrated probability of medication intensification[21] and adherence[15,35,36] to match the observed SBP and medication number in the published intensive SBP goal arm[1]
ACC/AHA Usual Care	• BP goal: <130/80 mmHg[9]<br>• Duration of intervention: N/A<br>• Physician visit frequency: according to usual care, dependent on BP control, individual characteristics, visit characteristics[10,15-17,34]<br>• BP measurement accuracy: assumed 1 BP measure per visit[10,15-17,50]<br>• Medication intensification and adherence: according to usual care, probability of medication intensification[15] and adherence[15,35,36]
Team-Based Care	• BP goal: <130/80 mmHg[9]<br>• Duration of intervention: 1 year<br>• Physician visit frequency: according to usual care, dependent on BP control, individual characteristics, visit characteristics[10,15-17,34]<br>• TBC non-physician visit frequency: once every 6 weeks for one year then stop[10]<br>• BP measurement accuracy: assumed 3 BP measure per TBC non-physician visit and 1 BP measure per physician visit[10,15-17,50]<br>• Medication intensification: TBC non-physician visit probability increased[10,17], physician visit probability according to usual care[10,15,17]<br>• Medication adherence: according to usual care, probability of medication adherence[15,35,36]; increased non-physician visit frequency and intensification decreased the probability of medication discontinuation
**Population**	25,000 individuals sampled with replacement from NHANES participants meeting SPRINT eligibility criteria

**Time Horizon and Perspective**	10 years and lifetime (age 100 years or death); healthcare sector perspective
**Model Type**	Discrete event simulation - microsimulation
**Primary Outcomes**	BP outcomes, fatal or non-fatal CVD events, CVD events-related mortality, and treatment-related SAEs
**Secondary Outcomes**	All-cause mortality, life years, healthy life years, and physician and non-physician visits

ACC – American College of Cardiology; AHA – American Heart Association; BP – blood pressure; CVD – cardiovascular disease; DBP – diastolic blood pressure; HF – heart failure; MI – myocardial infarction; JNC 7 – Seventh Report of the Joint National Committee on Prevention, Detection, Evaluation, and Treatment of High Blood Pressure; NHANES - National Health and Nutrition Examination Survey; PCP – primary care provider; SBP – systolic blood pressure, SPRINT – Systolic Blood Pressure Intervention Trial; TBC – Team-Based Care.

Notes: This table compares the assumptions and inputs used for the simulations of the four simulated hypertensive management strategies, including variability in BP treatment goals, treatment intensification, patient medication adherence, PCP and non-PCP visit, intervention durations.

Table S3. Model Inputs.

Model Input	Mean	SD	Min	Max	Distribution Type	Source
**BP Changes with Treatment**						
**SBP reduction with antihypertensive treatment**						Law et al.[33], Law et al.[32]
Per quarter-standard dose						
Mean SBP reduction at 150 mmHg	6.700	0.281	6.149	7.251	Gamma	
Coefficient of reduction per mmHg difference in pre-treatment SBP	0.078	0.020	0.040	0.116	Gamma	
Per half-standard dose						
Mean SBP reduction at 154 mmHg	9.100	0.357	8.400	9.800	Gamma	
Coefficient of reduction per mmHg difference in pre-treatment SBP	0.100	0.025	0.050	0.150	Gamma	
Per standard dose						
Mean SBP reduction at 154 mmHg	10.900	0.357	10.200	11.600	Gamma	
Coefficient of reduction per mmHg difference in pre-treatment SBP	0.120	0.030	0.061	0.179	Gamma	
**DBP reduction with antihypertensive treatment**						Law et al.[33], Law et al.[32]
Per quarter-standard dose						
Mean DBP reduction at 90 mmHg	3.700	0.306	3.100	4.300	Gamma	
Coefficient of reduction per mmHg difference in pre-treatment DBP	0.088	0.022	0.045	0.131	Gamma	
Per half-standard dose						
Mean DBP reduction at 97 mmHg	5.500	0.421	4.675	6.325	Gamma	
Coefficient of reduction per mmHg difference in pre-treatment DBP	0.110	0.028	0.056	0.164	Gamma	
Per standard dose						
Mean DBP reduction at 97 mmHg	6.500	0.421	5.675	7.325	Gamma	
Coefficient of reduction per mmHg difference in pre-treatment DBP	0.130	0.033	0.065	0.195	Gamma	
**BP change due to regression to the mean, by baseline BP**						Bryant et al.[50]
SBP						
<120 mmHg	4.060	3.440	0.000	13.000	Normal	
120-129 mmHg	3.050	1.440	0.000	5.000	Normal	
130-139 mmHg	0.250	0.250	-2.000	2.000	Normal	

Model Input	Mean	SD	Min	Max	Distribution Type	Source
140-149 mmHg	-1.780	0.620	-4.000	0.000	Normal	
150-159 mmHg	-4.570	2.180	-10.000	0.000	Normal	
≥160 mmHg	-9.140	2.800	-14.000	0.000	Normal	
DBP						
<70 mmHg	2.190	1.850	0.000	7.000	Normal	
70-79 mmHg	0.610	0.290	0.000	1.000	Normal	
80-89 mmHg	-0.380	0.370	-3.000	0.000	Normal	
90-99 mmHg	-3.110	1.080	-7.000	0.000	Normal	
≥100 mmHg	-4.990	1.880	-9.000	0.000	Normal	
**Percent of possible BP reduction achieved with proportion of antihypertensive doses taken as directed***						Bryant et al.[50]
Polynomial regression coefficients						
Intercept	0.001	-	-	-	-	
1st-degree	-0.135	-	-	-	-	
2nd-degree	-0.479	-	-	-	-	
3rd-degree	24.244	-	-	-	-	
4th-degree	-60.897	-	-	-	-	
5th-degree	59.584	-	-	-	-	
6th-degree	-23.813	-	-	-	-	
7th-degree	2.494	-	-	-	-	
**Office Visit Frequency**						
**Physician visits**						
Usual care weeks between visits when BP controlled, by age						Medical Expenditure Panel Survey
50-59 years	48.676	27.553	2.000	102.680	Gamma	
60-69 years	44.224	26.537	2.000	96.236	Gamma	
70-79 years	40.881	24.972	2.000	89.827	Gamma	
≥80 years	36.413	21.343	2.000	78.244	Gamma	
Usual care weeks between visits when BP uncontrolled	27.575	10.413	2.000	47.567	Gamma	Turchin et al.[34], Bellows et al.[15]
Usual care change in weeks between visits when BP uncontrolled						
Age (per year)	-0.147	0.012	-0.117	-0.164	Normal	
Change in SBP since last visit (per mmHg increase)	-0.052	0.009	-0.074	-0.039	Normal	

Model Input	Mean	SD	Min	Max	Distribution Type	Source
Change in DBP since last visit (per mmHg increase)	-0.056	0.008	-0.065	-0.035	Normal	
Antihypertensive medication added at visit	-2.080	0.199	-2.470	-1.690	Normal	
White race	-1.000	0.487	-1.950	-0.040	Normal	
Female	-0.560	0.411	-1.390	0.220	Normal	
Last visit was with primary care provider	-2.900	0.309	-3.510	-2.300	Normal	
SPRINT Intensive weeks between visits, by follow up time						Wright et al.[1]
<3 month	4	-	4	24	-	
≥3 month	12	-			-	
**TBC non-physician visits**						Bryant et al.[10]
Weeks between visits, first year only	6	-	4	24	-	
**BP Measurement Accuracy**						
**Measurement error, by total number of visits and measurements per visit****						Kronish et al.,[51] Bryant et al.[17]
SBP						
1 visit with 1 measurement	0.000	8.100	-	-	Normal	
1 visit with ≥3 measurements	0.000	6.200	-	-	Normal	
2 visits with 1 measurement	0.000	5.940	-	-	Normal	
2 visits with ≥3 measurements	0.000	4.390	-	-	Normal	
≥3 visits with 1 measurement	0.000	5.000	-	-	Normal	
≥3 visits with ≥3 measurements	0.000	3.650	-	-	Normal	
DBP						
1 visit with 1 measurement	0.000	5.450	-	-	Normal	
1 visit with ≥3 measurements	0.000	4.370	-	-	Normal	
2 visits with 1 measurement	0.000	3.900	-	-	Normal	
2 visits with ≥3 measurements	0.000	3.120	-	-	Normal	
≥3 visits with 1 measurement	0.000	3.180	-	-	Normal	
≥3 visits with ≥3 measurements	0.000	2.550	-	-	Normal	
**Antihypertensive Medication Adherence**						
Usual care and TBC pill-taking adherence						Claxton et al.[35], Vrijens et al.[36]
1 class	0.900	0.070	0.681	0.963	Beta	
2 classes	0.845	0.057	0.700	0.927	Beta	
3 classes	0.823	0.055	0.712	0.932	Beta	
≥4 classes	0.747	0.053	0.669	0.881	Beta	
SPRINT intensive pill-taking adherence during intervention						

Model Input	Mean	SD	Min	Max	Distribution Type	Source
First year						
1 class	0.990	0.907	0.968	0.996	Beta	
2 classes	0.985	0.906	0.970	0.993	Beta	
3 classes	0.982	0.906	0.971	0.993	Beta	
≥4 classes	0.975	0.905	0.967	0.988	Beta	
Subsequent years						
1 class	0.940	0.442	0.809	0.978	Beta	
2 classes	0.907	0.434	0.820	0.956	Beta	
3 classes	0.894	0.433	0.827	0.959	Beta	
≥4 classes	0.848	0.432	0.801	0.929	Beta	
Probability of antihypertensive medication discontinuation within one year of initiation	0.430	0.049	0.340	0.535	Beta	Bellows et al.[15]
**Antihypertensive Medication Intensification When BP is Uncontrolled**						
Usual care probability, physician						Bellows et al.[15]
First medication when untreated						
SBP ≥20 mmHg above goal	0.333	0.032	0.313	0.440	Beta	
SBP <20 mmHg above goal	0.208	0.026	0.207	0.310	Beta	
Subsequent medications	0.130	0.033	0.065	0.195	Beta	
SPRINT Intensive probability during intervention, physician						Zheutlin et al.[21], calibrated
First year	0.483	0.007	0.468	0.498	Beta	
Subsequent years	0.208	0.007	0.199	0.216	Beta	
TBC probability during intervention, non-physician						Bryant et al.[10]
130-134 mmHg	0.375	-	0.130	0.650	-	
135-139 mmHg	0.675	-	0.130	0.900	-	
140-149 mmHg	0.713	-	0.333	1.000	-	
≥150 mmHg	0.950	-	0.333	1.000	-	
**Medication-Related Adverse Events**						
Probability of intolerable adverse events						Law et al.[32], Law et al.[33], NHANES
1 class	0.005	0.002	0.001	0.011	Beta	
2 classes	0.009	0.005	0.003	0.021	Beta	
3 classes	0.016	0.008	0.005	0.035	Beta	
4 classes	0.023	0.011	0.007	0.050	Beta	
5 classes	0.030	0.014	0.009	0.064	Beta	
Probability of serious adverse events						Xie et al.[22],

Model Input	Mean	SD	Min	Max	Distribution Type	Source
≤2 classes	0.009	0.001	0.006	0.010	Beta	Wright et al.[1]
>2 classes	0.013	0.002	0.011	0.017	Beta	
Probability serious adverse event is fatal, by age						NIS, Wright et al.[1], Bress et al.[2]
18-44 years	0.004	0.001	0.004	0.007	Beta	
45-64 years	0.011	0.003	0.005	0.017	Beta	
65-84 years	0.019	0.007	0.003	0.031	Beta	
≥85 years	0.031	0.010	0.015	0.053	Beta	
**CVD Events and Non-CVD Death**						
**Heart failure**						
Probability of concurrent HF with MI						McManus et al.[37], Spencer et al[38]
50-54 years	0.121	-	-	-	-	
55-64 years	0.200	-	-	-	-	
65-74 years	0.290	-	-	-	-	
75-84 years	0.387	-	-	-	-	
≥85 years	0.477	-	-	-	-	
Probability of HF within 1 year of an MI	0.016	0.003	0.015	0.017	Beta	Blumer et al.[39], Steg et al.[40], calibrated
Annual Probability of HF from 1 to 5 years after an MI	0.009	-	-	-	-	Gerber et al.[41], calibrated
Concurrent MI and HF 30-day case fatality rate, by age						Spencer et al.[38]
50-54 years	0.146	-	-	-	-	
55-64 years	0.170	-	-	-	-	
65-74 years	0.220	-	-	-	-	
75-84 years	0.266	-	-	-	-	
≥85 years	0.296	-	-	-	-	
HF 30-day case fatality rate, by age						NIS, Krumholz et al.[42]
50-54 years	0.042	-	-	-	-	
55-64 years	0.060	-	-	-	-	
65-74 years	0.078	-	-	-	-	
75-84 years	0.082	-	-	-	-	
≥85 years	0.144	-	-	-	-	
**Risk of incident CHD**						NHLBI Pooled Cohorts Study
Age	0.102	-	-	-	-	
Non-Hispanic Black	-0.198	-	-	-	-	

Model Input	Mean	SD	Min	Max	Distribution Type	Source
Former smoker	0.184	-	-	-	-	
Current smoker	0.460	-	-	-	-	
Cigarettes per day	0.006	-	-	-	-	
SBP	0.013	-	-	-	-	
Diabetes	0.596	-	-	-	-	
HDL-C	-0.016	-	-	-	-	
LDL-C	0.006	-	-	-	-	
eGFR	-0.006	-	-	-	-	
Current smoker * age	-0.014	-	-	-	-	
SBP * age	-0.0003	-	-	-	-	
Diabetes * age	-0.001	-	-	-	-	
HDL * age	0.0003	-	-	-	-	
LDL * age	-0.0002	-	-	-	-	
**Risk of non-ischemic HF**						NHLBI Pooled Cohorts Study
Age	0.131	-	-	-	-	
Non-Hispanic Black	0.522	-	-	-	-	
Former smoker	0.054	-	-	-	-	
Current smoker	0.294	-	-	-	-	
Cigarettes per day	0.795	-	-	-	-	
SBP	0.015	-	-	-	-	
Diabetes	0.013	-	-	-	-	
HDL-C	0.631	-	-	-	-	
LDL-C	-0.003	-	-	-	-	
eGFR	-0.011	-	-	-	-	
Non-Hispanic Black * age	-0.027	-	-	-	-	
SBP * age	-0.0002	-	-	-	-	
Diabetes * age	-0.014	-	-	-	-	
**Risk of non-CVD death**						NHLBI Pooled Cohorts Study
Age	0.099	-	-	-	-	
Non-Hispanic Black	0.350	-	-	-	-	
BMI	-0.098	-	-	-	-	
BMI^2	0.001	-	-	-	-	
Former smoker	0.232	-	-	-	-	
Current smoker	0.688	-	-	-	-	
Cigarettes per day	0.019	-	-	-	-	
Diabetes	0.019	-	-	-	-	
eGFR	-0.005	-	-	-	-	

Model Input	Mean	SD	Min	Max	Distribution Type	Source
Non-Hispanic Black * age	-0.012	-	-	-	-	
BMI^2 * age	0.00001	-	-	-	-	
Diabetes * age	-0.010	-	-	-	-	
**Risk of incident stroke**						NHLBI Pooled Cohorts Study
Age	0.142	-	-	-	-	
Non-Hispanic Black	0.479	-	-	-	-	
Current smoker	0.601	-	-	-	-	
SBP	0.020	-	-	-	-	
Diabetes	0.650	-	-	-	-	
HDL-C	-0.005	-	-	-	-	
LDL-C	0.002	-	-	-	-	
eGFR	-0.004	-	-	-	-	
Non-Hispanic Black * age	-0.023	-	-	-	-	
SBP * age	-0.0005	-	-	-	-	
Diabetes * age	-0.015	-	-	-	-	
Current smoker * age	-0.013	-	-	-	-	
**Risk adjustment**						
Relative risk of CVD events per 10-mmHg decrease in SBP						Ettehad et al.[31]
CHD	0.830	-	0.780	0.880	Lognormal	
HF	0.720	-	0.670	0.780	Lognormal	
Stroke	0.730	-	0.680	0.770	Lognormal	
Hazard ratio of mortality risk with HF						Gerber et al.[41]
CVD mortality	2.94	-	2.41	3.58	Lognormal	
Non-CVD mortality	2.10	-	1.74	2.55	Lognormal	
**Health Adjustments to Life Years**						
**Chronic**						
Age	-0.0007	-	-	-	-	Sullivan et al.[24]
Obese	-0.050	-	-	-	-	
Number of comorbidities	-0.055	-	-	-	-	
Number of comorbidities^2	0.003	-	-	-	-	
Other CHD	-0.041	-	-	-	-	
Cardiac arrest	-0.019	-	-	-	-	
HF	-0.064	-	-	-	-	
MI	-0.041	-	-	-	-	
Stroke	-0.052	-	-	-	-	
Diabetes	-0.035	-	-	-	-	

Model Input	Mean	SD	Min	Max	Distribution Type	Source
ESRD	-0.060	-	-	-	-	
Hyperlipidemia	-0.005	-	-	-	-	
Hypertension	-0.025	-	-	-	-	
**Acute Events**						
*Acute CVD event****						
Cardiac arrest	0.0948	0.0150	0.0648	0.2340	Beta	Moran et al.,[28] Moran et al.,[27] Murray et al.[25]
MI	0.095	0.015	0.065	0.234	Beta	
Other CHD	0.094	0.015	0.064	0.124	Beta	
Stroke	0.136	0.015	0.106	0.166	Beta	
HF	0.100	0.015	0.070	0.130	Beta	King et al.,[26] Bress et al.[2]
Adverse events						
Intolerable****	0.200	0.030	0.170	0.230	Beta	CVDPM
Serious†	0.100	0.030	0.080	0.130	Beta	Bress et al.[2]

BMI – body mass index, BP – blood pressure, CHD – coronary heart disease, CVD – cardiovascular disease, DBP – diastolic blood pressure, eGFR – estimated glomerular filtration rate, ESRD – end-stage renal disease, HDL-C – high-density lipoprotein cholesterol, HF – heart failure, LDL-C – low-density lipoprotein cholesterol, MI – myocardial infarction, NHANES – National Health and Nutrition Examination Survey, NHLBI – National Heart, Lung, and Blood Institute, NIS – National Inpatient Sample, SBP – systolic blood pressure, SD – standard deviation, SPRINT – Systolic Blood Pressure Intervention Trial, TBC – team-based care.

*Expected BP reduction depends on the number of antihypertensive medications; the actual achieved reduction depends on imperfect pill taking execution which varies by the number of antihypertensive medications. The polynomial function multiplies the coefficient by the pill-taking adherence to the power indicated (e.g., 2nd-degree is multiplied by pill-taking adherence squared) to estimate the percent of possible BP reduction achieved.
**Increasing the number of visits and measurements per visit increases the diagnostic accuracy of measured BP. At each visit, the difference between the patient's underlying BP and the BP measured at the visit is estimated by sampling from normal distributions with a mean of 0 (i.e., no difference between underlying and measured BP) and a standard deviation that decreases with more visits and more measurements per visit.
***Applied for up to four weeks
****Applied for two days
†Applied for up to two weeks

Table S4. Performance of Model Calibration to SPRINT Outcomes.

Outcome	Calibration Target Mean (95% CI)	Simulated Mean (95% UI)	% in Range*
SBP Achieved (mean mmHg)			
Standard	134.6 (132.1 – 137.1)	134.2 (132.6 – 135.9)	99.5%
Intensive	121.5 (119.0 – 124.0)	120.4 (118.7 – 122.1)	94.5%
Number of Medications (mean)			
Standard	1.8 (1.3 – 2.3)	1.8 (1.7 – 1.9)	100.0%
Intensive	2.8 (2.3 – 3.3)	2.9 (2.8 – 3.1)	100.0%
Primary Composite CVD (per 100 person-years)			
Standard	2.2 (2.0 – 2.4)	2.3 (2.1 – 2.5)	89.0%
Intensive	1.7 (1.5 – 1.9)	1.7 (1.5 – 2.0)	86.0%
CVD Mortality (per 100 person-years)			
Standard	0.4 (0.3 – 0.6)	0.4 (0.3 – 0.4)	97.0%
Intensive	0.3 (0.2 – 0.4)	0.3 (0.3 – 0.4)	96.0%
MI (per 100 person-years)			
Standard	0.8 (0.7 – 0.9)	0.8 (0.7 – 0.9)	83.0%
Intensive	0.7 (0.6 – 0.7)	0.6 (0.5 – 0.8)	78.0%
Stroke (per 100 person-years)**			
Standard	0.5 (0.4 – 0.5)	0.5 (0.4 – 0.5)	88.0%
Intensive	0.4 (0.4 – 0.5)	0.3 (0.2 – 0.4)	2.0%
HF (per 100 person-years)			
Standard	0.7 (0.6 – 0.8)	0.7 (0.5 – 0.8)	77.0%
Intensive	0.4 (0.4 – 0.5)	0.5 (0.4 – 0.5)	47.0%
ACM (per 100 person-years) – primary analysis**			
Standard	1.4 (1.3 – 1.6)	1.4 (1.3 – 1.6)	97.0%
Intensive	1.0 (0.9 – 1.2)	1.4 (1.2 – 1.6)	2.0%
ACM (per 100 person-years) – secondary analysis***			
Standard	1.4 (1.3 – 1.6)	1.4	-
Intensive	1.0 (0.9 – 1.2)	1.0	-
SAE (per 100 person-years)			
Standard	0.8 (0.7 – 0.9)	0.7 (0.5 – 0.8)	74.0%
Intensive	1.5 (1.3 – 1.7)	1.5 (1.2 – 1.7)	84.5%

ACM – all cause mortality, CVD – cardiovascular disease, HF – heart failure, MI – myocardial infarction, SAE – serious adverse events, SBP – systolic blood pressure, 95% CI – 95% confidence interval, 95% UI – 95% uncertainty interval.

*This column shows the proportion of the 200 probabilistic iterations that had simulated means within the calibration target range, defined as the 95%CI from SPRINT for each outcome.
**In the primary analysis, the relative risk per 10-mmHg SBP reduction was applied for stroke from meta-analysis and the intensive arm was not calibrated to better match the results of SPRINT; no benefit was assumed for SBP lowering on non-CVD death.
***In the secondary analysis, a relative risk per 10-mmHg SBP reduction was applied to non-CVD death to replicate the ACM rate from SPRINT. The model was only run a single time for each of these analyses; thus, no 95%UI or percent in range are displayed.

Note: This table compares the simulation outputs to the calibration targets from SPRINT. The mean and 95% UI were calculated by running 200 probabilistic iterations in which the model was run repeatedly when randomly sampling input parameters from prespecified statistical distributions.

Table S5. Performance of Model for SBP at One Year with Team-Based Care vs. Usual Care.

Outcome	Calibration Target Mean (95% CI)	Simulated Mean (95% UI)	% in Range*
SBP (mmHg), TBC vs. usual care	10.5 (4.8 – 16.2)	11.8 (10.7 – 12.8)	100.0%

SBP – systolic blood pressure, TBC – team-based care, 95% CI – 95% confidence interval, 95% UI – 95% uncertainty interval.

*This column shows the proportion of the 200 probabilistic iterations that had simulated means within the calibration target range, defined as the 95%CI from the published meta-analysis.

Note: This table compares the simulated SBP at one year with TBC vs. usual care with a published meta-analysis of TBC with non-physician team members who can titrate antihypertensive medications. The mean and 95% UI were calculated by running 200 probabilistic iterations in which the model was run repeatedly when randomly sampling input parameters from prespecified statistical distributions.

Table S6. Projected Mean Number of Hypertension-related Provider Visits at 10 Years.

Outcome	JNC 7 Usual Care	SPRINT Intensive	ACC/AHA Usual Care	Team-Based Care
**SPRINT-representative**				
Physician office visits	18.9 (17.5 – 20.4)	31.6 (29.7 – 33.7)	22.8 (20.7 – 25.1)	16.9 (15.6 – 18.3)
Incremental	Ref.	12.7 (11.7 – 13.7)	3.9 (3.0 – 4.9)	-2.0 (-2.7 – -1.6)
TBC visits	-	-	-	7.2 (7.2 – 7.2)
**SPRINT-eligible US Adults**				
Physician office visits	18.5 (17.3 – 19.8)	33.3 (30.7 – 36.1)	24.4 (22.1 – 27.1)	18.3 (16.8 – 20.0)
Incremental	Ref.	14.9 (13.3 – 16.7)	5.9 (4.7 – 7.5)	-0.2 (-0.7 – 0.5)
TBC visits	-	-	-	7.2 (7.2 – 7.2)

ACC – American College of Cardiology; AHA – American Heart Association; JNC 7 – Seventh Report of the Joint National Committee on Prevention, Detection, Evaluation, and Treatment of High Blood Pressure; SPRINT – Systolic Blood Pressure Intervention Trial; TBC – Team-Based Care.

Note: The table shows the projected mean number of healthcare provider visits at which hypertension could be managed over 10 years with each hypertension management strategy in a population weighted to resemble SPRINT participants and weighted to resemble all SPRINT-eligible US adults. The results are presented as the mean and 95% uncertainty interval calculated by running 200 probabilistic iterations in which the model was run repeatedly when randomly sampling input parameters from prespecified statistical distributions.

Table S7. Scenario Analysis When Model is Calibrated to Replicate All-Cause Mortality Benefits in SPRINT.

Outcome	JNC 7 Usual Care	SPRINT Intensive	ACC/AHA Usual Care	Team-Based Care
**Events per 1000 individuals at 10 years***				
Prevented deaths	Ref.	40	13	33
Prevented CVD-related deaths	Ref.	10	4	8
**Events per year in US adults****				
Prevented deaths	Ref.	118,600	38,900	87,300
Prevented CVD-related deaths	Ref.	15,900	4,000	7,300
**Survival and healthy life years in US adults*****				
Increased life years	Ref.	2.4	0.9	1.4
Increased healthy life years	Ref.	1.9	0.7	1.2

ACC – American College of Cardiology; AHA – American Heart Association; CVD – cardiovascular disease; JNC 7 – Seventh Report of the Joint National Committee on Prevention, Detection, Evaluation, and Treatment of High Blood Pressure; SPRINT – Systolic Blood Pressure Intervention Trial; SAEs – serious adverse events.

*The simulated population was weighted to resemble SPRINT participants and is the projected number of events prevented vs. JNC 7 Usual Care per 1000 individuals over 10 years.
**The simulated population was weighted to represent all SPRINT-eligible US adults and is the estimated number of events prevented vs. JNC 7 Usual Care per year.
***The simulated population was weighted to represent all SPRINT-eligible US adults and is the estimated increase in survival and healthy life years over the remaining lifetime (age 100 years or death).

Note: The table shows how the results of the model changed when it was calibrated to reproduce the all-cause mortality results observed in both arms of SPRINT by including a relative risk per 10-mmHg SBP lowering applied to the risk of non-CVD death. Only a single iteration of the model was run for each scenario.

Table S8. Scenario Analysis When Simulating Hypertension Management with Usual Care Targeting SPRINT Intensive BP Goals.

Outcome	Usual Care Targeting BP Goal			SPRINT Intensive	Team-Based Care
	JNC 7	ACC/AHA	SPRINT Intensive		
**Events per 1000 individuals at 10 years***					
CVD events	242	229	210	184	201
Coronary heart disease	133	129	119	108	112
Stroke	54	48	42	35	41
Heart failure	55	52	49	41	48
SAEs	44	55	82	78	48
Death from any cause	179	175	175	168	167
CVD-related deaths	52	48	46	40	43
SAE-related deaths	1	1	2	1	1
**Events per year in US adults****					
CVD events	597,300	568,100	508,800	467,600	514,300
Coronary heart disease	308,100	292,200	273,000	257,600	275,700
Stroke	149,900	139,800	118,000	101,300	116,500
Heart failure	139,300	136,100	117,800	108,700	122,100
SAEs	59,400	90,300	136,800	137,200	82,900
Death from any cause	382,700	377,400	368,500	359,100	361,200
CVD-related deaths	113,900	108,700	97,700	90,300	101,000
SAE-related deaths	1,000	1,500	2,100	2,700	1,900
**Survival and healthy life years in US adults*****					
Life years	20.6	20.8	21.0	21.2	21.0
Healthy life years	15.8	16.0	16.2	16.4	16.2

ACC – American College of Cardiology; AHA – American Heart Association; BP – blood pressure; CVD – cardiovascular disease; JNC 7 – Seventh Report of the Joint National Committee on Prevention, Detection, Evaluation, and Treatment of High Blood Pressure; SPRINT – Systolic Blood Pressure Intervention Trial; SAEs – serious adverse events.
*The simulated population was weighted to resemble SPRINT participants and is the projected number of events per 1000 individuals over 10 years. **The simulated population was weighted to represent all 18.1 million SPRINT-eligible US adults and is the estimated number of events per year. ***The simulated population was weighted to represent all 18.1 million SPRINT-eligible US adults and is the estimated survival and healthy life years over the remaining lifetime (age 100 years or death).
Note: The table shows the results when simulating usual care management processes for the entire time horizon and targeting the SPRINT Intensive BP goal (<120/90 mmHg). Only a single iteration of the model was run and the other strategies from the primary analysis are included from this iteration for comparison.

Figure S1. BP-CVDPM Schematic.

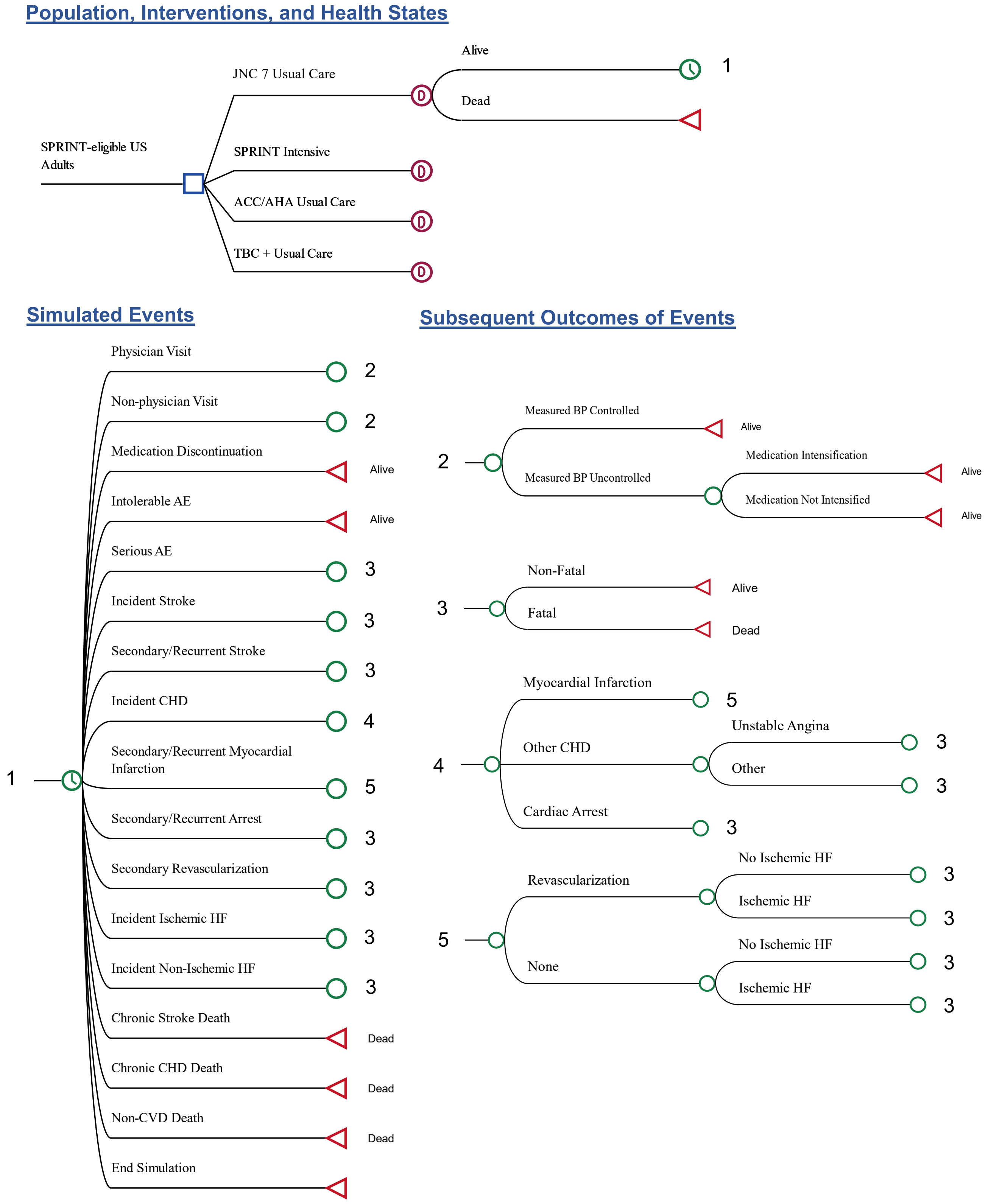

ACC – American College of Cardiology; AE – adverse event; AHA – American Heart Association; BP – blood pressure; BP-CVDPM – Blood Pressure Control-Cardiovascular Disease Policy Model; CHD – coronary heart disease; CVD – cardiovascular disease; HF – heart failure; JNC 7 – Seventh Report of the Joint National Committee on Prevention, Detection, Evaluation, and Treatment of High Blood Pressure; SPRINT – Systolic Blood Pressure Intervention Trial.

Notes: The figure shows a schematic of the discrete event simulation version of the BP-CVDPM. The model structure is the same for each arm, but only the JNC 7 Usual Care arm is shown for convenience. During each analysis, individuals are simulated to receive each strategy being compared. The purple "D" node represents the discrete event node, which, in this model, is where the possible health states of alive or dead are represented. The green "clock" node represents the time node, the point at which the model determines the next event that will happen given an individual's current characteristics. After an event occurs, the model determines any subsequent outcomes, shown by the chance nodes (green circles) and terminal nodes (red triangles). When an individual reaches the end of a pathway, the model assesses the outcomes experienced during that event and they return to the health state indicated. If they are still alive and the simulation hasn't ended, the model updates their characteristics and determines the time to their next event. Chronic stroke is death within one year of a stroke.

Figure S2. Distribution of Baseline Systolic Blood Pressure in SPRINT and NHANES Participants Weighted to Resemble SPRINT Participants.

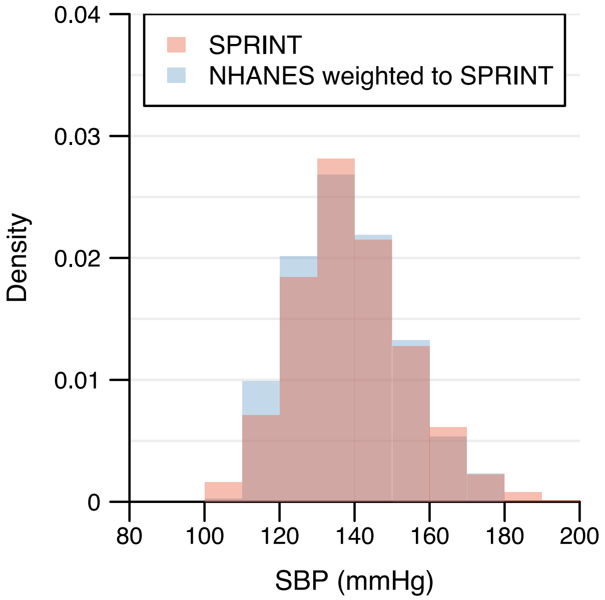

NHANES – National Health and Nutrition Examination Survey, SBP – systolic blood pressure, SPRINT – Systolic Blood Pressure Intervention Trial.

Note: The figure shows histograms of the published baseline SBP from SPRINT and the simulated population of SPRINT-eligible NHANES participants weighted to resemble the baseline characteristics of SPRINT participants.[1,43]

Figure S3. Performance of Model for SBP Achieved Compared with SPRINT Outcomes.

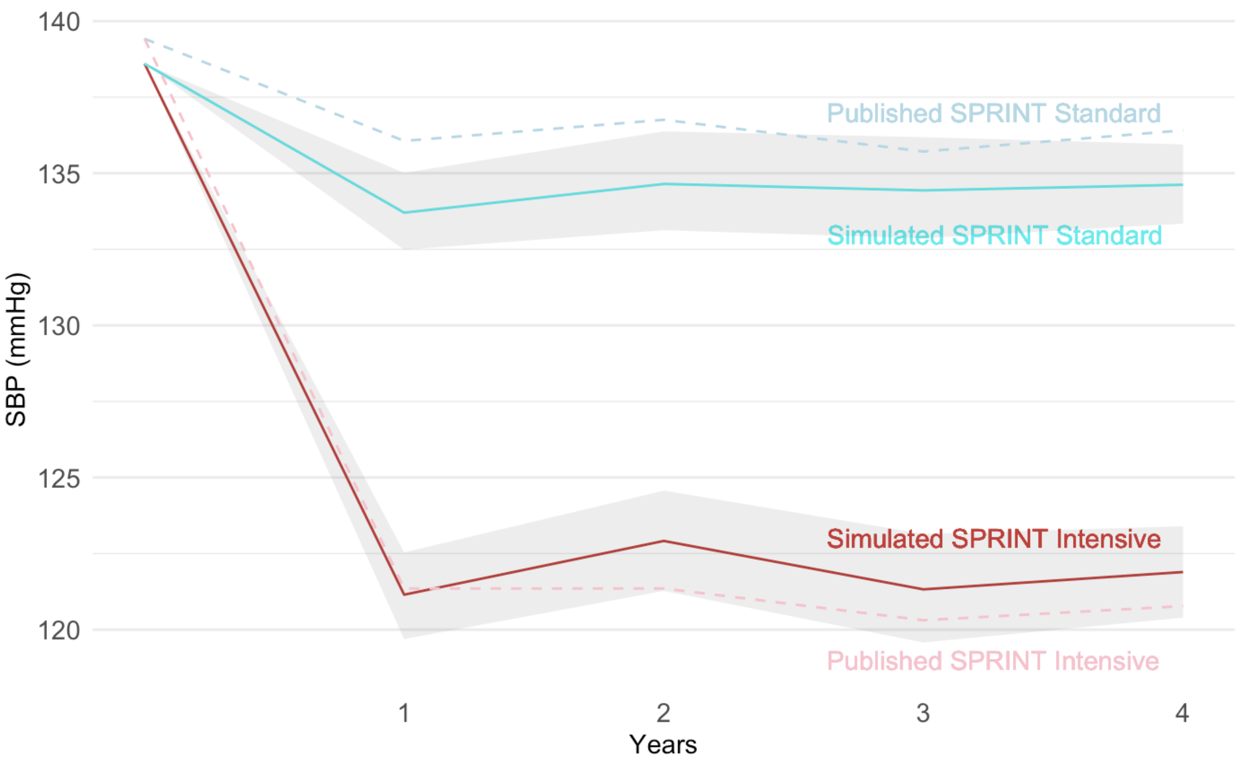

SBP – systolic blood pressure, SPRINT - Systolic Blood Pressure Intervention Trial.

Note: The figure shows the SBP changes over four years projected by the model compared with the published SBP changes in SPRINT. The lines represent the mean and shaded regions the 95% uncertainty interval, which were calculated by running 200 probabilistic iterations in which the model was run repeatedly when randomly sampling input parameters from prespecified statistical distributions.

Figure S4. Cumulative Incidence of SAEs over 10 years.

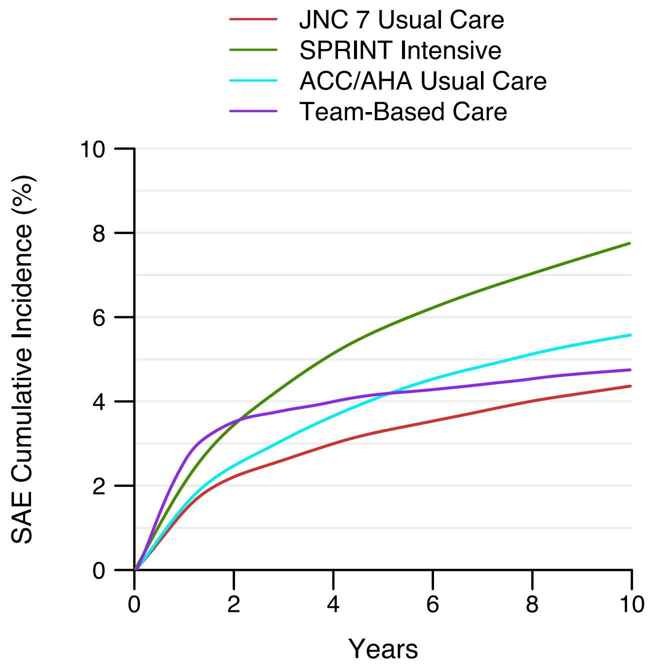

ACC – American College of Cardiology; AHA – American Heart Association; JNC 7 – Seventh Report of the Joint National Committee on Prevention, Detection, Evaluation, and Treatment of High Blood Pressure; SAE – serious adverse event; SPRINT – Systolic Blood Pressure Intervention Trial.

Notes: The figure shows the projected cumulative incidence of SAEs over 10 years with each simulated strategy in the primary analysis.

Figure S5. Sensitivity Analysis Independently Varying Hypertension Care Process Parameters.

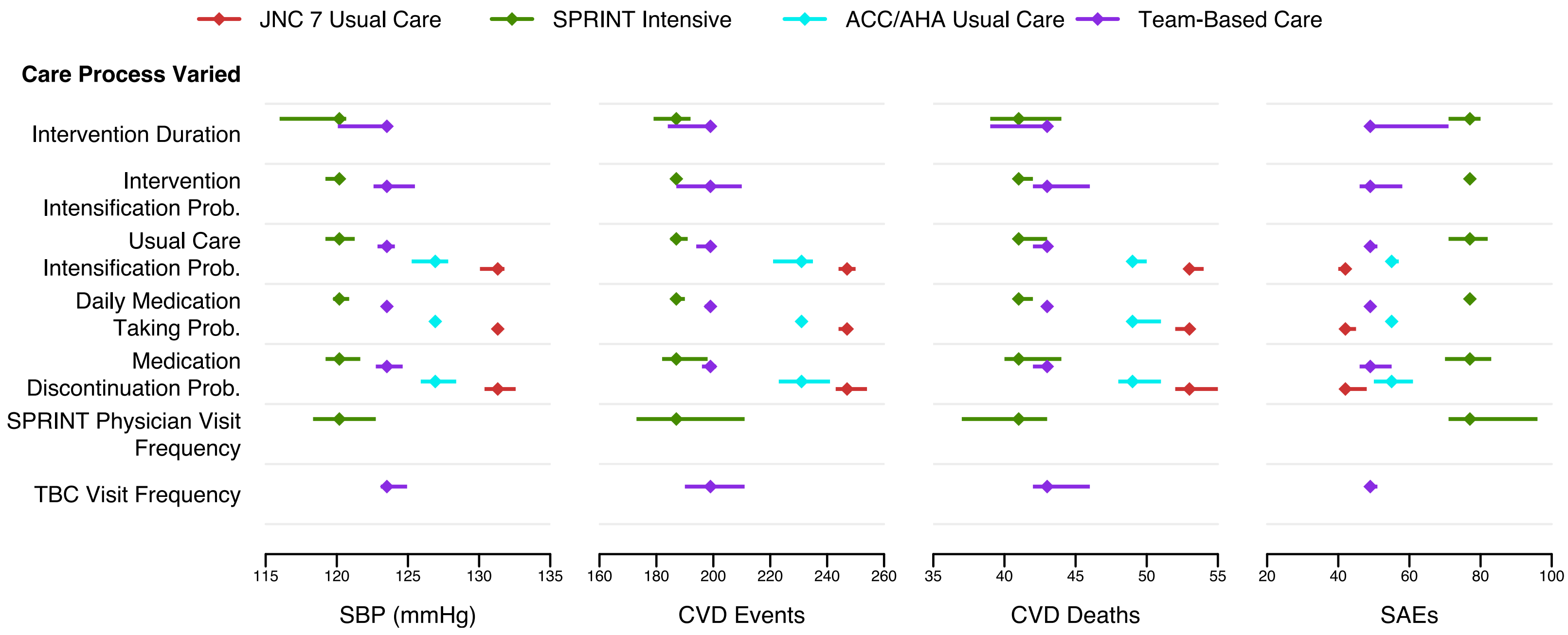

ACC – American College of Cardiology; AHA – American Heart Association; CVD – cardiovascular disease; JNC 7 – Seventh Report of the Joint National Committee on Prevention, Detection, Evaluation, and Treatment of High Blood Pressure; prob. – probability; SAE – serious adverse event; SBP – systolic blood pressure; SPRINT – Systolic Blood Pressure Intervention Trial; TBC – team-based care.

Note: The figure shows how independently varying hypertension care process parameters across plausible ranges changes the mean SBP achieved and CVD events, CVD-related deaths, and SAEs per 1000 individuals at 10 years. The base-case results are represented by the diamonds; results are not shown when the parameter did not contribute to a strategy (e.g., only team-based care is affected when varying the visit frequency of team-based care).
